# Bibliographical Notices

**Published:** 1854-06

**Authors:** 


*Types of Mankind: or Ethnological Researches based upon the ancient Monuments, Paintings, Sculptures and Crania of Races; and upon their natural, geographical, philological and biblical history; illustrated by selections from the inedited papers of* Samuel George Morton, M. D., late President of the Academy of Natural Sciences at* Philadelphia, and by additional contributions from Prof. L. Agassiz, LL. D., W. Usher, M. D., and Prof. H. S. Patterson, M. D. By J. C. Nott, M. D. of Mobile, Alabama, and Geo. R. Gliddon, formerly U. S. Consul at Cairo. 8vo. pp. 738. Lippincott, Grambo & Co. Philadelphia, 1854.

This work, after the preface, opens with a memoir of the late Dr. Samuel George Morton, written under the influence of an affectionate recollection of the subject, by Henry S. Patterson, M. D. This notice of Dr. Morton, has reference chiefly to his ethnological studies, but does not fail to mention his labors in various departments of learning. It contains some observations touching Dr. Morton's opinions, not stated in the biographical notices previously published; but beyond this, Dr. Patterson has not added any thing to our knowledge of the public life of our late much esteemed fellow-citizen. The result of Dr. Morton's ethnological studies, was a conviction that the human race is not the offspring of a single pair, but of several pairs; an opinion of great importance, whether considered in relation to its social, philosophical or religious relations. It is not within the limits we have prescribed for ourselves, to discuss this or any other opinion stated or advocated in the work before us.

Dr. Morton had few competitors in the field of ethnology on this side of the Atlantic, and is admitted to have been a leader among the most distinguished in this department of knowledge. A reference to his published works, and the unrivalled collection of crania made by him, will prove him to have been a zealous, careful and accurate student, and assure us that his conclusions are entitled to profound respect. The principal works of Dr. Morton on ethnological subjects, are his "Crania Americana," published October, 1839, and his "Crania Ægyptiaca," which consists of essays read before the American Philo-

* The title of the Academy should be "the Academy of Natural Sciences of Philadelphia."—Ed.

sophical Society, in the years 1842 and 1843, and published in a volume in 1844.

This biographical notice of Dr. Morton is followed by an essay of twenty pages, entitled, "Sketch of the natural provinces of the animal world, and their relation to the different types of man," By Louis Agassiz; in which it is alleged that not only the several races of men, but all the groups of animals and plants, constituting distinct faunæ and floræ had distinct and separate origins, one for each of the eight realms, under which all animals are locally classed. They are the 1. Arctic; 2. Asiatic; 3. European; 4. American; 5. African; 6. East Indian or Malayan; 7. Australian, and 8. Polynesian realms. The sketch is accompanied by a map, and pictorial illustration representing the fauna of each realm, as far as may be, by figures of animals of the class mammalia, with a portrait of a man of each realm at the head of each series. This picture has the merit of having been composed or drawn in the Library of the Academy of Natural Sciences of Philadelphia, under the eye of Mr. Gliddon; it has all the value this circumstance can give to such an effort.

Next follows an introduction by J. C. Nott, M. D., of a dozen pages, in which Ethnology is defined; and Mr. Pritchard and the Book of Genesis are very fiercely assailed. Chapter I. is on the "Geographical distribution of animals, and the races of men." Chapter II. is devoted to general remarks on types of mankind. The terms type and species, are regarded as synonymous, and Dr. Morton's definition of species—"a primordial organic form,"—is accepted, but it is not very well determined what circumstances are included in the term form; is color necessarily an element of specific characteristics? If color alone is enough to distinguish species, it might be urged by over-zealous naturalists, those who are distinguished by a prurience for making and describing new species, that white cats are not of the same species as black cats, although of the same genus.

It is not necessary to state the contents of the several chapters.

The object of the work is to prove that the population of the earth is not derived from a single pair, as stated in the Book of Genesis, and admitted by Cuvier and other naturalists of great learning. Cuvier says,—"Although the promiscuous intercourse of the human species, which produces individuals capable of propagation, would seem to *demonstrate* its unity, certain hereditary peculiarities of conformation are observed, which constitute what are termed *races*." But Cuvier regarded these "hereditary peculiarities," not as constituting difference of species, but only varieties of the same species. There are certain peculiarities, very remarkable in themselves, which run through families, such as supernumerary figures, toes, &c., but these are not considered to be specific characteristics. Nor is size in itself, without anatomical difference, enough to constitute species.

The value of a work of this kind, depends very much upon the nature of the evidence, and the character of the witnessess brought forward to sustain the broad assertions made by its authors. Mr. Agassiz is distinguished by his extended learning in all the departments of Natural History, but a plain man needs something more, even from him, than assertion, to induce him to abandon belief in the Mosaic account of the creation. His paper is a mere generalization of his notions, in the shape of assertion.

Dr. Morton rests his belief chiefly upon the difference of cubic capacity in the skulls of the inhabitants of different regions of the earth, who existed at different periods. He has drawn all his inferences from the measurement of at most 900 human skulls, of all nations and races, ancient and modern, living and extinct. The data these furnish are certainly interesting, and afford remarkable degrees of comparison; but it may well be doubted whether they are enough for a demonstrated conclusion. What might be the character, for instance, of the present inhabitants of Philadelphia, if it should be deduced two hundred or a thousand years hence, by some learned Japanese or denizen of some other far off region, after measuring a hundred and fifty skulls taken, hap-hazard, from the cemetery at Laurel Hill, or any other burial place of the city or county? Yet, in some instances, forty or even fewer skulls have been enough to furnish Dr. Morton with satisfactory conclusions, as to the cranial characteristics of a nation.

Another kind of evidence adduced is found in the rude effigies of ancient tombs. The ethnologists implicitly rely upon the accuracy of the cranial portraiture in figures, which, in almost every respect, are marvellously inaccurate in their anatomical representations of different parts of the body. As an illustration, look at figures 163 and 164, page 248, and believe, if possible, that such shoulders, bodies or extremities belonged to yellow, black or white races at any period of the world's history? What would be our opinion of a philosopher who would take any portrait in any historical picture of modern times, as a means of studying the phrenological developments of the person represented? Yet, one of our ethnologists goes further than this. Speaking of the "Asiatic conquests of Ramses II," "within the fourteenth century, B. C." Dr. Nott says, p. 158, "Mr. Birch's detailed account of this important historical document is accompanied by colored drawings, in which the victories of that monarch over various Asiatic and African races are represented with amazing truthfulness and spirit." Dr. Nott surely should indicate how he knows said representations to be truthful. It is not believed by philosophers generally that poets, painters and sculptors are the most accurate or the most reliable witnesses to establish absolute fact, or even historical truth, which, of the varieties of truth, is not the most trustworthy in scientific or physical investigations.

It is doubtful whether the following assertion of Dr. Nott will bear the test of scrutiny: "Not an individual breathes, in whose veins flows blood acknowledged to be 'royal,' but traces his or her genealogy to this Norman colossus, William the Conqueror!" If this be true absolutely, it might serve in the argument for the unity of the origin of the human race; if this William be in fact the sole progenitor of all living kings and princes of Christendom who are not usurpers; if he be the original fountain of all legitimate royalty, why not assign, with equal propriety, all humanity to a single pair, for there are not greater differences among Christian kings and princes than between the races of men.

As another specimen of deduction and creation of facts, if such an expression can be tolerated, take the following: "From an investigation of the successive growths of the cypress forests around that city, [New Orleans,] the stumps of which are still found *at different depths, directly overlying each other;* [a monstrous overlying it must be, too,] from the great size and age of these trees, and from the remains of Indian bones and pottery found below the roots of some of these stumps, he [Dr. Dowler] arrives at the following conclusion: 'From these data it appears that the human race existed in the delta [of the Mississippi] more than 57,000 years ago; and that ten subterranean forests, and the one now growing, will show that an exhuberant flora existed in Louisiana more than 100,000 years anterior to these evidences of man's existence.'"

Whether this "conclusion" has been a result of the Baconian method of induction, and is better vouched for than the Mosaic history and chronology, is a question we need not now waste time to discuss.

The inedited manuscript of the late Dr. Morton occupies 28 pages of the volume. The tone of the following extract is so entirely opposite to anything expressed by the authors of the work, dedicated to his memory, that it is worthy of special attention.

Dr. Morton says, "We earnestly maintain that the preceding views are not irreconcilable with the Sacred Text, nor inconsistent with Creative Wisdom, as displayed in the other kingdoms of nature. On the contrary, they are calculated to extend our knowledge and exalt our conceptions of Omnipotence. By the simultaneous creation of a plurality of original stocks, the population of the earth became not an accidental result, but a matter of certainty. Many and distant regions which, in accordance with the doctrine of a single origin, would have remained for thousands of years unpeopled and unknown, received at once their allotted inhabitants; and these, instead of being left to struggle with the vicissitudes of chance, were from the beginning adapted to those varied circumstances of climate and locality which yet mark their respective positions upon the earth."

There is in this some respect exhibited towards the Mosaic account, which Dr. Nott and Mr. Gliddon seem disposed to regard derisively; but there is also shown the littleness of man's mental power and vain assumption in his contemplation of the work of the Creator. What train of reasoning led Dr. Morton to fancy that God had left any part of the creation "to struggle with the vicissitudes of chance?" Can it be imagined for a moment that what we denominate the vicissitudes of chance are not under the control and direction of those fixed laws which an All wise hand has placed over all things, both physical and spiritual? There is no reason to suppose that there is any thing in the creation left to chance or hap-hazard contingencies.

The propriety of publishing the crude or unfinished labors of a literary or scientific man is, to say the least, questionable. Had Dr. Morton lived to revise the paragraph above quoted, it is probable he would have modified the expression of opinion in this instance, and would have refrained from assigning motives to the Creator for any part of his works. This conjecture is based upon a statement by our authors that Dr. Morton found reason to change views published by him in his Crania Ægyptiaca.

On page 215 we are told that "Morton's *Crania Ægyptiaca* issued in 1844; at which day the discoveries of Lepsius were in progress, but not published; at the same time that the works of Rossellini, Champollion, Wilkinson, &c.—then the best sources of information respecting the monuments—did not extend, with the exception of some meagre materials, to the XIIth dynasty (by all three scholars then supposed to be the XVIIth,) beyond the XVIIIth, or about 1600 B. C."

On page 218 we are informed that Dr. Morton "made important modifications in some of his opinions," and again, page 229, "the Doctor himself had so far changed his opinions as *to feel assured* that the primordial Egyptians were not an Asiatic, but an aboriginal population, indigenous to the Nile-land, &c."

Can Messrs. Gliddon and Nott give us any guarantee that Dr. Morton would not have changed his opinions again, had he lived up to this time?

"Geology and palæontology, in connexion with human origins," is treated of by William Usher, M. D., of Mobile, in forty-five pages.

Hybridity of animals and the comparative anatomy of Races are treated by Dr. Nott.

Believing the entire work to be conceived and executed in an unphilosophical spirit, with a view to putting forth a special plea for one side of a question, instead of a simple and earnest endeavor to establish simple truth, which cannot be inconsistent with itself, no matter whether it be manifested physically or by spiritual revelation, it is no more worthy of critical and systematic examination than is Tom Paine's Age of Reason, although it has less merit as a literary effort than the work of that renowned deist.

All that the authors have said, in spite of their many curious and interesting statements, leads to no definite conclusion. They have failed to establish any one point they have ventured to assert. Their work abounds in repetitions; and were they to recommit their materials to the process of thought and reflection, it is very possible that Dr. Nott or Mr. Agassiz might state every fact and argument adduced in one half the space.

The self-sufficiency, scientific assumption, and wordiness of Mr. Gliddon, place him far beyond the reach of our critical ability. It is our misfortune to believe that, in spite of his association with Agassiz, Morton, Usher and Nott, he will never be considered as authority for any thing in positive science, though in the field of conjecture he may always retain a conspicuous position.

The publishers, Messrs. Lippincott, Grambo & Co., are entitled to praise for the mechanical and artistic qualities of the volume, which are in all respects worthy of their well deserved reputation.

---

### *Transactions of the American Medical Association.*

(Continued.)

*Report of the Committee on Medical Education,* N. S. Davis, M.D. *Chairman.*

The general subject is considered under three heads, viz: the medical periodicals of our country; the medical publications, including monographs and books; and the best means of elevating the character, and extending the usefulness of our national medical literature.

During the year from April, 1852, to April, 1853, there were published in the United States, twenty eight medical periodicals; of which four were issued quarterly; six bi-monthly; fifteen monthly; two semi-monthly; and one weekly.

The arrangement of these journals is alike in most respects, embracing original articles, cases, &c., reviews and bibliographical notices; selections from foreign and domestic sources, and editorials.

The editorials and selections are favorably spoken of, the former, we are informed, being generally written with candor and liberality, and the latter being judiciously chosen, and presenting matter of practical interest. As regards the original department of our journals, "many of the articles are of no value, for want of fulness of observation and completeness of detail. Others present isolated facts of interest. And a comparatively small proportion are related with such minuteness and accuracy, and in such numbers, as to afford deduction of great practical and scientific value."

Faulty and worthless as great portions of it is, yet the Reporter is of opinion, judging from the comments of former committees, that a decided improvement has taken place in the original department of our journals, during the last two years; and justly, we think, attributes the melioration to the influence of the association, and its auxiliary societies.

The review and bibliographical department, with some gratifying exceptions, which are noticed, is stated to be "meagre and unsatisfactory in the extreme, consisting of little more than a copy of the title-page of the book, purporting to have been examined, with a single paragraph embodying a very general expression of opinion, and that almost always of a commendatory character."

"A comprehensive and impartial survey of the medical writings of our country, whether contained in periodicals, monographs or books, has induced us to refer their defects, directly or indirectly, to four primary sources. These are, *first*, deficiency of preliminary education; *second*, the absence of a clear and definite perception of the fundamental principles of physiology and pathology; *third*, defective modes of investigation; and, *fourth*, too much haste in the preparation of matter for the press."

It would be interesting, had we the time, to compare these opinions with those of Dr. La Roche, in his admirable introductory letter to Chas. D. Meigs, M. D., in his late work on "Pneumonia."

Dr. La Roche's intimate acquaintance with our own and foreign medical literature, entitles his opinion to great weight. We shall merely state that his views, regarding the ignoble condition of our medical literature, and his explanation of the causes which have produced such a state, agree, in many respects, with those given by Dr. Davis.

To remove the evils and imperfections complained of, which may be all traced to deficient education, or indirectly to an imperfect comprehension of the tasks undertaken, our great reliance must be placed in "the creation of such a sentiment throughout the profession, as will not only restrain individual members from encouraging uneducated young men to enter the ranks of the profession; but it will, also, develope a nobler idea of what a physician should be, and with it an appetite for a purer, more philosophical, and comprehensive professional literature. The means for creating such a sentiment, are already in existence and active operation. They embrace the several faculties connected with the Medical Colleges, the conductors of our medical periodicals, and the active members of our national, State and local medical organizations."

*On the agency of the Refrigeration, produced by upward radiation of Heat, as an existing cause of disease.*

The agency of nocturnal terrestrial radiation in the production of disease, has received little or no attention from writers on Hygeine. It is the object of this Report to indicate the importance and proper method of guarding ourselves in sickly places and during epidemic periods, against the injurious and depressing influences due to the refrigeration produced by this cause. "That much sickness is thus produced, must, we think, be apparent to any one who knows that on calm and clear nights, bodies exposed to the open sky are liable to a reduction of 10° or 20° of temperature through the effect of radiation. That is to say, whilst a thermometer suspended in the open air, under a tent or other shelter, may indicate a temperature of 80°, a person outside, exposed to the clear sky, may be subjected to a refrigerating agent, calculated to produce a reduction of temperature sufficient to bring down the thermometer to 65° or ever 60°. The effects of a refrigeration much less than here stated, would, if long kept up, tend to disturb the healthy functions of the animal economy, especially in those who are peculiarly sensitive to changes of temperature." The slighest covering, even the flimsy protection afforded by a pocket-handkerchief, or a bush from a tree, will suffice to intercept the view of the sky, and thus prevent radiation, with its mischievous consequences."

The perusal of this paper has afforded us much gratification. The study of the application of natural laws to the preservation of health and consequent longevity of man, still remains a most extensive field, deserving of the investigation of the highest intellect. It gives us much pleasure to commend the report, as a most valuable and suggestive paper.

*On the Results of Surgical Operations in Malignant Diseases.*

The object of Dr. Gross's paper is to exhibit the present state of opinion, in our own country and abroad, regarding the subject of his report. Under Part I. he notices, 1, the nature, objects and difficulties of the inquiry, the term malignant being applied to scirrhus, encephaloid, colloid and melanosis.

In the first division he considers, 1st, the nature, objects, and difficulties of the enquiry. 2. The origin of malignant diseases. 3. Their hereditary nature. 4. Their latency. 5. Circumstances contraindicating surgical interference. 6. Reproductive tendency after operation. 7. General rules respecting the manner of conducting excision. 8. Treatment after operation.

In the second division, we have, 1st, facts and observations contributed by J. Mason Warren, M. D., of Boston. Dr. Warren relates the history of twenty cases of cancerous tumors, and from the review of these, and the general impression of the results of his own practice, feels himself justified in drawing the following conclusions:

1. That in a certain number of cases, cancerous tumors, once removed, do not again return.

2. That in a certain other number, the patient, after immunity for a longer or shorter period, has a return of disease, requiring a second operation, which sometimes proves successful.

3. That in a great proportion of cases, the disease, after removal, returns either in the original wound, in its neighborhood, or in some internal organ.

4. That in consequence of the relief from pain during the operation, afforded by anæsthetic agents, and for reasons mentioned above, it is better to remove the tumor, provided the health be not much impaired and the disease yet remains local. For, in case of return, it generally reappears in a milder form, and the patient is saved from the loathsome character it assumes when allowed to proceed unrestrained to a fatal termination.

To this is added a table of recorded operations in Dr. Warren's hospital and private practice.

2. Table of results of surgical operations in malignant diseases, by Frank H. Hamilton, M. D.

3. Facts and observations contributed by different surgeons and gleaned from American and European authors.

The third division treats of cancer of particular organs, in relation to the results of surgical operations.

A large part of this branch of the subject is devoted to cancer of the breast. Cancer of the eye, of the gums and jaws, of the lip, of the penis, of the testicle, of the uterus, of the anus and rectum, and of the bones, are also described. From a consideration of the facts and statements presented, certain general conclusions are arrived at, for which we refer our readers to the report, merely stating that Dr. Gross has no confidence in any operation for malignant diseases, except the cancroid varieties.

In the March number of the Edinburgh Monthly Journal, there is an excellent review (by Dr. Bennett, we presume,) of Mr. Paget's lectures on Surgical Pathology, in connexion with M. Velpeau's Treatise on Diseases of the Breast, and Mr. Robert Druitt's work on the Modern Philosophy of Cancer. It is therein stated that M. Velpeau has demonstrated that MM. Lebert, Robin, Broca, and Follin have been frequently wrong in attributing malignancy, from microscopical observations, to tumors which were, in point of fact, innocent, and that other tumors, "maintained by them to be innocent, rapidly returned after extirpation, in various parts of the body, destroyed the patient and were consequently malignant." M. Velpeau has also "shown that tumors undoubtedly cancerous, both in a surgical and histological point of view, may be excised with the most perfect success." Several cases of this latter kind are referred to by the reviewer, and some are given us, from which we must draw the inference "that true cancerous tumors, both hard and soft, not merely said to be so as the result of experience, but *proved* to be so by careful histological examination, are admitted and described by one of the first surgeons of the day, to be capable of being successfully extirpated from the body, and causing a restoration of perfect health from at least three to six years, and in a few cases even longer. But Mr. Paget says that neither he nor Lebert have yet met with a case in which recurrence was delayed beyond eight years; and as to ultimate cure, he adds, "without saying it is impossible, it is so highly improbable, that a hope of its occurring in any single case cannot be reasonably entertained." "It is of the utmost importance, therefore, in reference to this question, that M. Velpeau should continue to watch his cases, because on him seems to depend the speedy solution of the great practical question, whether a tumor *proved* to be a cancer, may or may not be permanently eradicated."

Respecting secondary tumors, M. Velpeau says that he has "cured, radically, women after three successive operations, and some others after the second." "M. Roux has succeeded in bringing about a permanent cure, after six returns of the cancer." The reviewer closes with the statement that our present notions on the malignancy of these affections "are faulty in the extreme, and a far more extended series of researches is required before we are warranted to approach, much less dogmatise on, morbid growths."

These statements, it will be seen, are entirely opposed to the opinions expressed by Dr. Gross in his general conclusions. The subject is a very perplexing one. The study of structure is however so much advanced, and there are so many men of high scientific attainments engaged in its investigation, that we have a right to expect that much of the discrepancy of opinion now existing, will, in the course of a few years, be dissipated on these momentous points.

(To be continued.)

---

*Elementary Chemistry: Theoretical and Practical.* By GEORGE FOWNES, P. H. D., &c. *Edited with additions by* ROBERT BRIDGES, M. D. Blanchard & Lea.

There are few tasks more difficult to complete successfully than that of preparing a good text book of Chemistry. To bring within a limited number of pages, all that is essential to a general knowledge of the science, to explain the laws that govern chemical affinity, to enumerate the elements and their inorganic compounds, and to give even but a passing glance at the most prominent objects in the unlimited field of organic chemistry, would seem to require indeed no ordinary powers. But when we find that of the space thus limited, one-fourth must be devoted to Physics, a subject having no necessary connection with Chemistry, and as capable of being studied, as well without the help of the latter, as with it, we would feel inclined to throw up the task, in despair of ever doing justice to the science we undertook to teach, or of giving even the faintest outlines of its infinitudes of matters.

On first taking up Fownes' Chemistry, we were disposed to view it as a mere epitome of the text books of others, but on comparing it with the American editions of Turner's and of Grahams', we found that, notwithstanding it is not more than half the size of these, it was in all respects equal to either in fulness of detail, and in systematic arrangement. This is in part due to the print being closer and smaller, but also in a greater degree to the condensed style, which without being, like Gmelin's Handbuch, for instance, so very terse, dry and statistical as to be useless as a text book, yet contains a great amount of matter in as little space as possible, without falling into the above mentioned extreme.

We never open any of the well known treatises on chemistry, without regretting that so little is said about Microscopical Chemistry. The microscope is now almost as necessary to the Chemist as the balance or the blow-pipe, not only in physiological but even in inorganic Chemistry. Although, in the examination of urine and in the detection of the crystallizable constituents or the various fluids of the body, &c. &c., it is indispensable, yet in every general treatise with which we are familiar, it is entirely omitted; and animal chemistry is almost entirely left to the Physiologist, to whose domain it does not more properly belong, than vegetable chemistry does to that of the Botanist. In regard to physiological chemistry, the work before us is certainly superior to any of the systematic treatises of general chemistry with which we are acquainted, containing better and more complete observations upon the fluids of the body; viz. blood, milk, urine, &c. &c., with microscopical views of the globules contained therein, directions for detecting sugar and albumen in urine; and woodcuts illustrating the most important of the urinary calculi. The limited size of the work prevents the above being treated very much in detail, but in the absence of works devoted especially to this branch, this volume cannot but prove of great utility to the student in physiological chemistry.

The last English edition of Fownes' Chemistry, produced under the superintendence of the distinguished chemists H. Bence Jones and A. W. Hoffman, appeared towards the end of 1852. When we state that the American edition has been edited by Dr. Bridges of this city, we need give no further assurance that the work contains all the new discoveries and theories of value known in this country at the date of publication, Oct. 1853. Much valuable information omitted in the English edition, has been supplied in this, in foot notes by the Editor. We notice, for instance, remarks on the antidotes for and detection of arsenious acid; the latest views on the nature of many organic compounds; the basic nature of glycerine, &c. &c. We much regret that the Editor of the American Edition was not able to insert an abstract of Liebig's admirable process of determining urea, phosphates and chlorides in urine, published in the Annalen der Chemie, March, 1853. To the physician who wishes to experiment upon the constituents of normal or diseased urine, its value cannot be too highly extolled.

In addition to previous remarks, we must draw attention to the fact, that this edition is the latest treatise on Chemistry, and in so progressive a science, every thing else being equal, the latest work is by far the best one.

---

*A Practical Treatise on Inflammation of the Uterus, its Cervix and Appendages, and on its Connexion with Uterine Disease.* By JAMES HENRY BENNETT, M. D., &c. Fourth American from the Third and revised London Edition. Philadelphia. Blanchard & Lea. 1853.

No writer of the present day, on the subject of uterine pathology, has acquired a higher reputation than Dr. Bennett. His first edition, published in 1845, drew the attention of the profession to the frequency of chronic inflammation of the neck of the uterus, as the cause of most of the affections to which that organ is subject. The value of the speculum for facilitating diagnosis, and for the application of remedies, was also pointed out and strongly insisted upon. His views on these points have been before the profession for many years, and have consequently far passed beyond that period of time in which criticism could in any way affect their reputation. The present edition has been carefully revised and rendered more complete by many additions.

*First Principles of Medicine.* By ARCHIBALD BILLING, M. D., A. M., F. R. S., &c. Second American from the revised and improved Fifth London Edition. Philadelphia. Lea & Blanchard. 1854.

Dr. Billing's "First Principles" may be safely recommended as a work containing much valuable and suggestive matter. Its worth has been highly appreciated, it having passed through five editions.

